# Aberrant Wnt signaling in multiple myeloma: molecular mechanisms and targeting options

**DOI:** 10.1038/s41375-019-0404-1

**Published:** 2019-02-15

**Authors:** Harmen van Andel, Kinga A. Kocemba, Marcel Spaargaren, Steven T. Pals

**Affiliations:** 1 Department of Pathology, Academic Medical Center, University of Amsterdam, Amsterdam, The Netherlands; 2 Lymphoma and Myeloma Center Amsterdam (LYMMCARE), Amsterdam, The Netherlands

**Keywords:** Cell signalling, Myeloma

## Abstract

Aberrant activation of Wnt/β-catenin signaling plays a central role in the pathogenesis of a wide variety of malignancies and is typically caused by mutations in core Wnt pathway components driving constitutive, ligand-independent signaling. In multiple myelomas (MMs), however, these pathway intrinsic mutations are rare despite the fact that most tumors display aberrant Wnt pathway activity. Recent studies indicate that this activation is caused by genetic and epigenetic lesions of Wnt regulatory components, sensitizing MM cells to autocrine Wnt ligands and paracrine Wnts emanating from the bone marrow niche. These include deletion of the tumor suppressor *CYLD*, promotor methylation of the Wnt antagonists *WIF1*, *DKK1, DKK3*, and *sFRP1*, *sFRP2*, *sFRP4*, *sFRP5*, as well as overexpression of the co-transcriptional activator BCL9 and the R-spondin receptor LGR4. Furthermore, Wnt activity in MM is strongly promoted by interaction of both Wnts and R-spondins with syndecan-1 (CD138) on the MM cell-surface. Functionally, aberrant canonical Wnt signaling plays a dual role in the pathogenesis of MM: (I) it mediates proliferation, migration, and drug resistance of MM cells; (II) MM cells secrete Wnt antagonists that contribute to the development of osteolytic lesions by impairing osteoblast differentiation. As discussed in this review, these insights into the causes and consequences of aberrant Wnt signaling in MM will help to guide the development of targeting strategies. Importantly, since Wnt signaling in MM cells is largely ligand dependent, it can be targeted by drugs/antibodies that act upstream in the pathway, interfering with Wnt secretion, sequestering Wnts, or blocking Wnt (co)receptors.

## Introduction

Multiple myeloma (MM) is a clonal expansion of malignant plasma cells in the bone marrow (BM) that is typically accompanied by paraproteinemia, lytic bone lesions, hypercalcemia, cytopenias, and renal failure. It is preceded by monoclonal gammopathy of undetermined significance (MGUS), an asymptomatic premalignant accumulation of clonal plasma cells that shares genetic features with MM [1]. In ~50% of MGUS and MM patients, recurrent translocations involving the immunoglobulin heavy chain (IgH) locus are found, which drive the expression of juxtaposed genes, including Cyclin D1 (*CCND1*,11q13), *FGF3/MMSET* (4p16.3), Cyclin D3 (*CCND3*, 6p21), *MAFC* (16q23), and *MAFB* (20q11). Most of the remaining cases are hyperdiploid, with recurrent trisomies of chromosomes 3, 5, 7, 9, 11, 15, 19, and 21 [2, 3]. Despite this heterogeneity, all these primary genetic events are believed to drive cell cycle entry by deregulating D-type cyclins, a unifying pathogenic event crucial for malignant transformation of plasma cells [4]. In addition, multiple secondary genetic and epigenetic abnormalities have been identified that drive MM progression. These include general hypomethylation, gene-specific hypermethylation, mutations in *KRAS*, *NRAS*, *BRAF*, and *P53*, genetic and epigenetic abnormalities in NF kappa B and Wnt pathway components, and genetic aberrations in the *MYC* family of oncogenes [2, 3, 5]. Despite the broad landscape of genetic and epigenetic abnormalities, virtually all MM tumors are strictly dependent on the BM microenvironment, or niche, for growth and survival [6, 7].

The MM microenvironment consists of various extra-cellular matrix components and cell types, including BM stromal cells, osteoblasts, osteoclasts, and endothelial cells. These cells secrete factors such as interleukin(IL)-6, insulin-like growth factor (IGF), hepatocyte growth factor (HGF) and a proliferation-induced ligand (APRIL), which collectively provide signals essential for growth and survival [6, 8]. Both normal and malignant plasma cells are highly decorated with the heparan sulfate proteoglycan (HSPG) syndecan-1, which facilitates communication with the BM niche by binding and presenting numerous secreted factors and promoting signal transduction and adhesion [9–11]. During disease progression, MM cells continuously interact with and shape the microenvironment to favor tumor growth. This disrupts BM homeostasis, resulting in cytopenias and lytic bone lesions. Interestingly, the canonical Wnt signaling pathway plays a dual role in the reciprocal interaction between MM cells and the BM niche: (I) the BM microenvironment facilitates aberrant activation of canonical Wnt signaling in MM cells, and thereby plays an important role in tumorigenesis; (II) MM cells secrete Wnt antagonists which contribute to the development of lytic bone lesions by impairing osteoblast differentiation. In this review, we examine the causes and biological consequences of aberrant Wnt signaling activity in MM cells and discuss possible strategies to target the Wnt pathway in MM.

## The Wnt signaling pathway

The Wnt cascade represents a highly conserved developmental signal-transduction pathway involved in a variety of cellular processes, including regulation of proliferation, cell-fate, migration, and cell polarity. There are 19 *Wnt* genes in the human genome which encode lipid-modified secreted glycoproteins, acting as ligands for their cognate Frizzled (FZD) receptors. Wnts are relatively unstable and insoluble due to their hydrophobic nature, which constrains long-range signaling. As a consequence, they act as typical niche or stem cell factors [12, 13]. The lipid modification of Wnt proteins involves covalent attachment of a palmitoyl group, appended by the palmitoyltransferase Porcupine (encoded by *PORC*), and is required for signaling and secretion [14, 15]. Wnt signals can be transduced in distinct ways; by a well-defined “canonical” Wnt/β-catenin pathway, or by either of two β-catenin independent “non-canonical” Wnt signaling cascades, designated the Wnt/PCP and Wnt/Ca^2+^ signaling pathway. Whereas some members of the Wnt ligand and Fzd receptor families are strictly dedicated to either canonical or non-canonical signaling, most Wnts and Fzds are promiscuously involved in both pathways. In this review, we will mainly focus on canonical Wnt signaling.

**Fig. 1:**
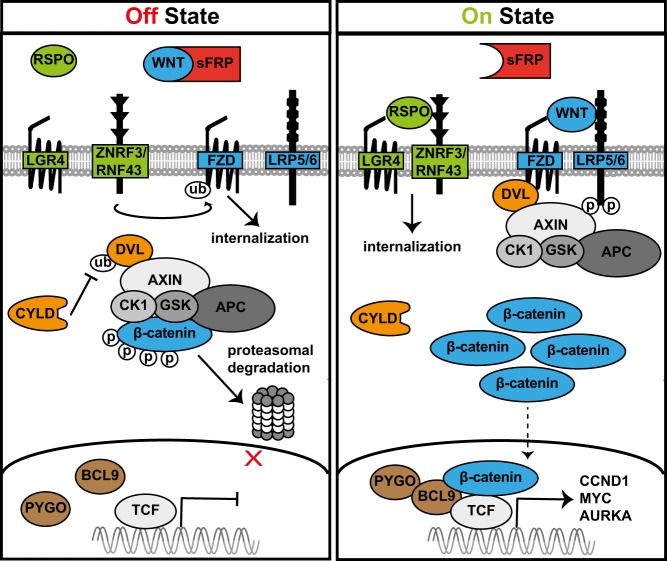
Schematic representation of canonical Wnt signaling. *Off state* (left panel): In the absence of Wnt ligands, β-catenin is continuously phosphorylated by a destruction complex that includes AXIN, APC, GSK3β, and CK1α, which marks it for proteasomal degradation. In addition, Wnt signaling is antagonized at multiple levels. First, the secreted Wnt inhibitors sFRP and DKK1 prevent activation of Wnt signaling by sequestering Wnt ligands or preventing LRP5/6 phosphorylation, respectively. Second, in the absence of LGR4/R-spondin signaling, the E3 ubiquitin ligases ZNRF3 and RNF43 antagonize Wnt activity by ubiquitinating Wnt (co)receptors, which induces internalization and subsequent degradation. Lastly, the deubiquitinase CYLD impairs intracellular signal transduction by removing Lys-63-linked polyubiquitin chains from the adapter protein Disheveled (Dvl), which decreases protein stability. *On state* (right panel): Binding of a Wnt ligand to its receptor Frizzled induces phosphorylation of the co-receptors LRP5/6, which forms a docking site for AXIN. Subsequent sequestration of AXIN disrupts the destruction complex and allows stabilization and nuclear translocation of non-phosphorylated β-catenin. In cooperation with the TCF/LEF family of transcription factors and the co-transcriptional activators Pygopus (PYGO) and BCL9, this orchestrates transcription of Wnt target genes. In addition, LGR4/R-spondin signaling facilitates signaling by Wnt ligands. Engagement of R-spondin to its receptor LGR4 induces internalization of ZNRF3/RNF43, thereby alleviating the negative regulatory role of these E3 ligases on Wnt receptor stability

Canonical Wnt signaling revolves around the stabilization and subcellular localization of the transcriptional coactivator β-catenin (Fig. 1). In the absence of Wnt ligands, β-catenin is continuously phosphorylated by a destruction complex that includes CK1α, AXIN, APC, and GSK3β, which marks β-catenin for ubiquitination and proteasomal degradation (Fig. 1, left panel). Engagement of a Wnt ligand to one of its cognate Fzd receptors initiates phosphorylation of the Wnt coreceptors LRP5/6, thereby creating a docking site for AXIN (Fig. 1, right panel). Subsequent translocation of AXIN to the plasma membrane disrupts the destruction complex and allows stabilization and nuclear translocation of non-phosphorylated β-catenin [12, 13]. In cooperation with TCF/LEF family transcription factors [16, 17] and other transcriptional coactivators, such as BCL9 [18], this orchestrates a transcriptional program comprising multiple targets including *MYC* and *CCND1* (encoding Cyclin D1) [19, 20].

In contrast to canonical Wnt signaling, non-canonical Wnt signaling is independent of LRP5/6 and β-catenin and plays an important role in regulating cell polarity, adhesion, and migration. In Wnt/PCP signaling, engagement of a Wnt ligand to a Fzd receptor results in activation of the small GTPase RhoA and downstream protein kinases, including Rho-associated protein kinase (ROCK), which regulates cytoskeletal dynamics by dictating the localization of structural proteins such as actin [21–23]. In the Wnt/Ca^2+^ pathway, binding of a Wnt ligand to its receptor results in the release of calcium ions from the endoplasmic reticulum (ER) via the activation of G-proteins, phospholipase C (PLC), and phosphodiesterase (PDE). In turn, elevation of intracellular calcium levels activates enzymes such as protein kinase C (PKC), resulting in altered cell motility [24].

Deregulation of canonical Wnt signaling plays a central role in malignant transformation and tumorigenesis in a variety of tissues. This is exemplified by the intestinal epithelium. In this archetypal Wnt-dependent tissue Wnt signaling is required for stem cell maintenance and, when hyperactivated, is the main driver of malignant transformation. Oncogenic Wnt signaling typically results from mutations in core pathway components such as APC, β-catenin, and AXIN, causing constitutive, ligand-independent signaling [25]. In addition, more recent evidence indicates that genetic and epigenetic alterations that increase the sensitivity of tumor cells to Wnt ligands can equally drive tumorigenesis. This is exemplified by genetic alterations in the ZNRF3/RNF43/R-spondin/LGR axis. ZNRF3 and RNF43 are homologous membrane-bound E3 ubiquitin ligases that attenuate signaling by inducing Wnt (co)receptor internalization. Their action is antagonized by leucine-rich repeat-containing G protein-coupled receptor (LGR)-family receptors in response to cognate R-spondin ligands [26–29] (Fig. 1). Inactivating mutations in *ZNRF3*/*RNF43*, as well as gene fusions of R-spondin causing overexpression, have recently been identified in a number of Wnt-driven tumors, including colorectal cancer (CRC) and pancreatic ductal adenocarcinoma [30–32]. These genetic alterations render the tumor cells highly sensitive to Wnt ligands by stabilizing Wnt (co-)receptors on the plasma membrane. Importantly, since Wnt activation in tumor cells carrying these mutations is still ligand-dependent, it can be targeted by drugs that interfere with Wnt secretion, sequester Wnts, or block Wnt receptors.

## Aberrant canonical Wnt signaling in MM cells

Initial evidence for a role of canonical Wnt signaling in the pathogenesis of MM came from a study by Derksen et al. [5] demonstrating that most primary MMs (pMMs) and human MM cell lines (HMCLs), unlike normal plasma cells, express non-phosphorylated, i.e. active, β-catenin in the nucleus. Moreover, inhibition of Wnt signaling by ectopic expression of a dominant negative TCF4 mutant (dnTCF4) in HMCLs suppressed proliferation, implying functional involvement of β-catenin/TCF-mediated transcription. Corroborating these results, other studies showed that siRNA [33] and shRNA [34] mediated silencing of β-catenin or treatment of HMCLs with the small molecule Wnt/β-catenin inhibitors PKF115-584 [35], AV-65 [36], BC2059 [37], CGK012 [38], and the FDA-approved Pyrvinium pamoate [39, 40] attenuated cell cycle progression, impaired tumor growth, and markedly increased apoptosis of HMCLs both in vitro and in vivo. In addition to attenuating proliferation, silencing of β-catenin increased the sensitivity of the tumor cells to multiple drugs used in the treatment of MM, in particular to lenalidomide [34, 41]. Gene-expression analysis after silencing of β-catenin [34] and treatment with PKF115-584 [35] and CGK012 [38] showed downregulation of the Wnt target genes *CCND1* (encoding cyclin D1) and *MYC*, which are known to play a key role in the pathogenesis of MM [4, 42, 43]. In addition to these established Wnt targets, several other genes involved in cell cycle regulation were also downregulated, including Aurora Kinase A (*AURKA)*, which was identified as an important effector of Wnt-driven proliferation in MM [34]. While these data collectively provided solid evidence for a functional role of canonical Wnt/β-catenin signaling in MM pathogenesis, the mechanisms underlying aberrant Wnt activation remained incompletely understood.

Oncogenic Wnt signaling is typically driven by tumor-cell intrinsic mutations in Wnt pathway components. For this review, we analyzed the mutational status of a number of Wnt pathway components in published MM datasets and identified pathogenic mutations in *APC*, *CTNNB1* (encoding β-catenin) and *RNF43* that are recurrently found in other Wnt-driven tumors (Table 1) [44–48]. Furthermore, a number of mutations were identified of which the pathogenic significance is currently unknown (Table 1). However, these Wnt pathway mutations occur at low frequency (3% overall), indicating that alternative mechanisms drive illegitimate Wnt activation in the majority of MMs. As discussed below, several lines of evidence indicate that paracrine Wnts emanating from the BM microenvironment and/or autocrine Wnts, in concert with loss of negative Wnt pathway regulators, fuel Wnt pathway activation in malignant plasma cells.

**Table 1:** Overview of genetic and epigenetic abnormalities in MM that induce or facilitate canonical Wnt activation

Gene	CDS change	AA change	Mutation type	Clinical significance	Recurrence	Frequency
Classic Wnt pathway mutations [44–48]						
APC	nr	S1465fs	Frameshift	Pathogenic	Recurrent in CRC	1/133
APC	6976C>G	R2326G	Missense	Likely pathogenic	Germline mutation in FAP	1/84
APC	718A>T	T240S	Missense	Unknown	Not previously reported	1/84
APC	5369G>A	R1790K	Missense	Unknown	Not previously reported	1/14
APC	497insA^a^	T166fs	Frameshift	Pathogenic	Not previously reported	nr
AXIN1	2089C>T	P697S	Missense	Likely pathogenic	Sporadic in HCC	1/203
AXIN2	944C>T	T315M	Missense	Likely pathogenic	Sporadic in GC	1/203
CTNNB1	nr	T41I	Missense	Pathogenic	Recurrent in CRC and PC	1/133
*RSPO/LGR4/RNF43/ZNRF3 mutations* [46]						
RSPO2	219G>T	E73D	Missense	Unknown	Not previously reported	1/203
RSPO2	257G>A	R86Q	Missense	Likely pathogenic	Recurrent in CRC and PC	1/203
RNF43	1813G>T	A605S	Missense	Unknown	Not previously reported	1/203
RNF43	1976delG	G659fs	Frameshift	Pathogenic	Recurrent in CRC and PC	1/203
ZNRF3	2419G>C	A807P	Missense	Unknown	Not previously reported	1/203
ZNRF3	68T>G	V23G	Missense	Unknown	Not previously reported	1/203
*Promotor methylation of secreted Wnt antagonists* [61, 62, 65]				*Abnormalities in LGR4* [63]		
**Gene**	**pMM**	**HMCLs**		mRNA overexpression	86% (30/35)	
APC	18% (9/50)	25% (1/4)				
WIF	22% (11/50)	50% (2/4)		*Abnormalities in CYLD* [44–48, 68–70]		
DKK1	33% (3/12)	67% (4/6)		Mutation	3% (22/744)	
DKK3	16% (8/50)	50% (2/4)		Deletion	17% (nr/nr)	
sFRP1	27% (33/123)	78% (7/9)		16q deletion	35% (40/114)	
sFRP2	52% (38/73)	56% (5/9)				
sFRP3/FRZB	nd	nd		*Abnormalities in BCL9* [69, 72, 78–80]		
sFRP4	7% (8/123)	56% (5/9)		mRNA overexpression	60% (38/64)	
sFRP5	6% (7/123)	67% (6/9)		Low *miR30* expression	60% (47/78)	
				1q gain	36% (192/530)	

Clinical significance and recurrence is based on previous publications on these specific mutations and/or occurrence of these mutations in the Catalog of Somatic Mutations in Cancer (COSMIC) database (www.cancer.sanger.uc.uk)

*CDS* coding DNA sequence, *AA* amino acid, *nr* not reported

^a^Patient with germline mutation in APC (attenuated form of familial adenomatous polyposis) that developed MM

## Non-canonical Wnt signaling in MM cells

The first evidence for a role of non-canonical Wnt signaling in MM came from a study by Qiang et al. [49], demonstrating that Wnt3a induces striking morphological changes in MM cells by regulating cytoskeleton dynamics. Whereas Wnt3a treatment resulted in activation of both canonical and non-canonical Wnt signaling, these morphological changes could be blocked by sequestration of Wnt ligands with sFRP-1 and inhibition of non-canonical Wnt signaling by a small molecule inhibitor of Rho-associated kinases, but not by DKK-mediated inhibition of canonical Wnt signaling. Moreover, activation of canonical Wnt signaling by the GSK inhibitor LiCl did not induce these morphological alterations. This indicated that the observed cellular changes were strictly mediated by non-canonical Wnt signaling. Subsequent studies from the same lab showed that activation of non-canonical Wnt signaling also enhanced migration and invasion of MM cells, which involved activation of RhoA, Disheveled (Dvl), and members of the PKC family, in particular PKCμ [50]. In addition, non-canonical Wnt signaling in MM cells enhanced integrin-mediated heterotypic cell adhesion, resulting in cell-adhesion-mediated drug resistance (CAM-DR) of MM cells to doxorubicin [51]. Collectively, these studies identified a role of non-canonical Wnt signaling in regulating migration, dissemination and CAM-DR of MM cells.

## Paracrine and autocrine Wnt signaling activation in MM

Wnts are produced in the BM microenvironment and have been implicated in hematopoietic stem cell (HSC) maintenance [52] and early B cell development [53]. Several studies have modeled paracrine signaling by activating Wnt signaling in MM cells with either exogenous Wnt ligands or the GSK3 inhibitors lithium chloride (LiCl) and 6-bromoindirubin-3-oxomine (BIO) in vitro. These treatments invariably induced β-catenin stabilization and Wnt reporter activity, indicating that MM cells are well equipped to respond to Wnt ligands. While several studies found that Wnt3a-conditioned medium, recombinant Wnt3a or GSK3 inhibition enhanced proliferation of HMCLs [5, 37], others did not observe this positive effect on tumor growth [54–56]. This might be explained by the different cell lines and compounds used and the promiscuous role of GSK3 in the regulation of multiple signal transduction pathways [57]. Moreover, it should be noted that accumulating evidence in CRC and HSC supports a ‘just-right’ model of Wnt activation, which proposes that each individual (tumor) cell has an optimal threshold of Wnt activation [58–60]. This implies that different levels of Wnt activation can have distinct, even opposite, cellular effects. Notably, most MMs display hallmarks of Wnt pathway activation in the absence of exogenously added Wnts, suggesting that autocrine Wnts might be important drivers. Indeed, gene expression analysis revealed expression of various Wnts (including Wnt 3, 4, 5A, 5B, 6, 7, 8A, 10A, 10B, 11, 14 and 16) in pMMs and HMCLs [5, 61, 62]. Furthermore, blocking Wnt secretion with the small molecule porcupine inhibitors IWP-2 and LGK974 [63] or opposing binding of Wnt ligands to Fzd with secreted Frizzled protein 1 (sFRP1) [62] decreased β-catenin stabilization, attenuated β-catenin/TCF-mediated transcription and impaired expansion of several HMCLs, confirming the existence of an autocrine Wnt signaling loop. Taken together, these data indicate that aberrant Wnt activation in malignant plasma cells can be fueled by autocrine and paracrine Wnt ligands and regulates proliferation and drug resistance of MM cells by inducing expression of Wnt target genes, such as *CCND1*, *MYC*, and *AURKA*. As discussed below, several genetic and epigenetic alterations have been identified in MM that reinforce this Wnt pathway activation, either by directly enhancing signaling by Wnt ligands or by facilitating intracellular signal transduction.

## Epigenetic silencing of Wnt antagonists, aberrant expression of LGR4 and HSPG facilitate ligand-induced Wnt signaling in MM

**Fig. 2:**
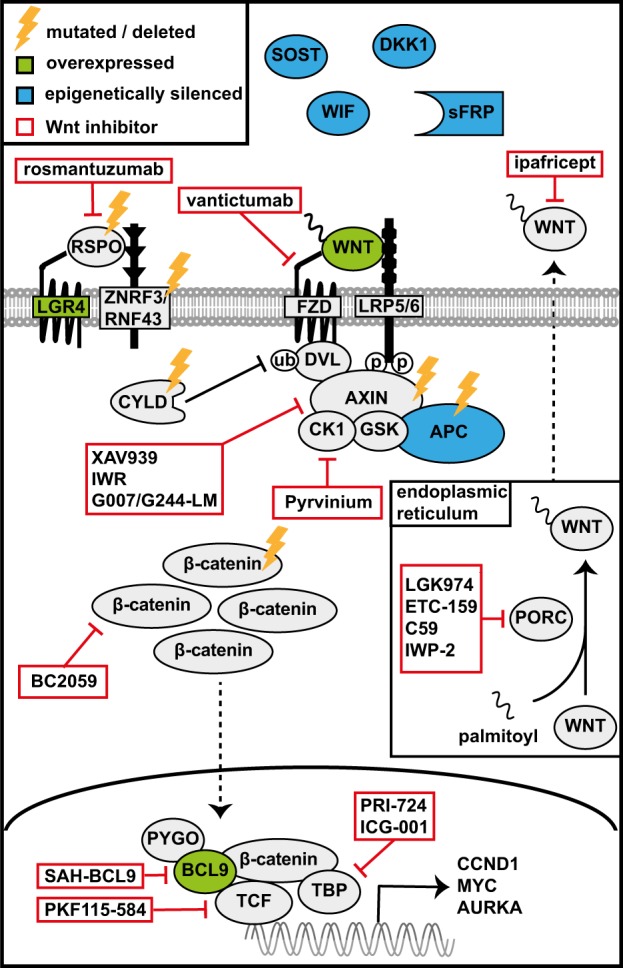
Schematic overview of genetic and epigenetic abnormalities in Wnt pathway components in MM and drugs that target canonical Wnt signaling. Aberrant canonical Wnt signaling pathway activation in MM cells is facilitated by epigenetic silencing (blue), overexpression (green), or mutation (yellow) of multiple Wnt pathway components. Notably, a variety of antibodies, small molecule, and peptides have been recently developed that target Wnt signaling at multiple distinct levels (red, see also Table 2)

Gene-specific promotor methylation is frequently observed in MM and can contribute to tumorigenesis by selectively and reversibly suppressing transcription of tumor suppressor genes, such as p16/INK4a (encoded by *CDKN2A*) [64]. Interestingly, epigenetic silencing of core Wnt pathway components and/or of secreted Wnt antagonists is frequently present in MM and likely plays a key role in facilitating Wnt activation. Hypermethylation of the promotor of *APC* or at least one of the secreted Wnt antagonists *WIF1*, *DKK1*, *DKK3*, and *sFRP1*, *sFRP2*, *sFRP4*, and *sFRP5* was detected in the majority of pMMs and HMCLs and was associated with advanced disease stage (Table 1 and Fig. 2). Over 60% of these patients displayed epigenetic silencing of multiple Wnt antagonist [61, 62, 65]. Reverting this promotor methylation by the demethylating agent decitabine resulted in re-expression of these antagonists and mitigated Wnt signaling, demonstrating that their silencing indeed facilitates Wnt activation in MM cells. In addition to epigenetic silencing of Wnt antagonists, aberrant expression of the R-spondin receptor LGR4 was identified as a cause of increased responsiveness of MM cells to Wnt ligands [63]. Analysis of LGR4 expression revealed it is expressed by the majority of MMs, but not by normal plasma cells (Table 1). LGR4 is transcriptionally regulated by IL-6/STAT3 signaling and allows MM cells to respond to R-spondins, which are produced in the BM microenvironment by cells of the osteoblast lineage [63]. Engagement of R-spondin by LGR4 alleviates Wnt (co)receptor internalization by ZNRF/RNF43, resulting in stabilization of Wnt (co)receptors and greatly increased sensitivity of tumor cells to Wnt ligands [26–29] (Fig. 1, right panel). Accordingly, while Wnt ligands alone only moderately activate Wnt signaling in HMCLs, addition of recombinant R-spondin or (pre)osteoblast-conditioned media dramatically enhances signaling by both autocrine and paracrine Wnt ligands. Moreover, silencing of LGR4 in HMCLs secreting autocrine Wnt ligands impaired proliferation in vitro, indicating functional involvement of LGR4 in mediating Wnt-driven tumorigenesis [63].

Interestingly, we recently reported that syndecan-1 (CD138) acts as a key mediator of both R-spondin/LGR4 signaling and signaling by Wnt ligands in MM [66]. Syndecan-1 is a HSPG that is strongly expressed by normal and malignant plasma cells and consists of a core protein decorated with covalently bound heparan sulfate chains that can promiscuously bind and present a variety of growth factors and cytokines [9–11]. Silencing of the enzyme exostosin 1 (*EXT1*), which is required for heparan sulfate chain synthesis, mitigated binding of both Wnts and R-spondins to MM cells and attenuated Wnt pathway activation. Moreover, silencing of *EXT1* attenuated the growth of MM tumors both in vitro and in vivo, which could be rescued by ectopic expression of a constitutive active β-catenin mutant or by c-MYC expression, indicating involvement of canonical Wnt signaling [66, 67].

## Deletion of the tumor suppressor CYLD, sumoylation of β-catenin, and overexpression the oncogene BCL9 hyperactivates Wnt signaling MM

The above described epigenetic alterations enhance the sensitivity of MM cells to Wnt ligands by affecting *membrane proximal* regulators of the Wnt signaling cascade. However, aberrant expression of intracellular Wnt pathway regulators CYLD and BCL9 can also strongly promote Wnt signaling in MM. Loss of the tumor suppressor *CYLD*, by gene deletion and/or inactivating mutations, is among the most common genetic abnormalities in MM and is associated with a poor prognosis [46, 47, 68–72] (Table 1). CYLD is a deubiquitinase that decreases stability of target proteins by specifically removing Lys63-linked polyubiquitin chains, and acts as a negative regulator of NF kappa B and Wnt signaling [73–75]. In Wnt signaling, CYLD targets and destabilizes the adapter protein Dvl, which impairs signal transduction (Fig. 1). Gene-expression analysis in pMM revealed a negative correlation between *CYLD* mRNA expression and the strength of a Wnt target gene signature. In addition, gene-set-enrichment analysis showed enrichment of Wnt pathway genes in *CYLD* low compared to *CYLD* high MM patients. These data suggest that loss of CYLD facilitates Wnt signaling in MM cells. Indeed, functional studies demonstrated that silencing of CYLD in MM cells enhances β-catenin stabilization and β-catenin/TCF-mediated transcription, while reintroduction of CYLD in MM cells that display a bi-allelic loss of the *CYLD* locus suppresses Wnt activity and impairs proliferation. Thus, loss of CYLD can promote Wnt pathway activation and drive proliferation in MM [71].

In addition to ubiquitination, sumoylation has also been implicated in the deregulation of canonical Wnt signaling in MM [76]. Increased protein sumoylation has been previously observed in MM and is associated with adverse patient outcome [77]. siRNA-mediated silencing of *SUMO1* in MM cells enhanced β-catenin phosphorylation and decreased β-catenin-mediated transcription. Moreover, this was accompanied by decreased proliferation and increased apoptosis of MM cells, which could be rescued by ectopic expression of a constitutive active β-catenin mutant. These results indicate that sumoylation of β-catenin can enhance Wnt/β-catenin signaling in MM cells [76]. Similar to CYLD deletion, aberrant expression of the transcriptional coactivator BCL9 has also been linked to hyperactivation of the Wnt pathway in MM. BCL9 functions by recruiting the transcriptional coactivator Pygopus to the β-catenin/TCF complex, which is required for transcription of Wnt target genes [18]. Gene-expression analysis revealed that BCL9 is overexpressed in approximately 60% of MMs, while BCL9 expression is negligible in normal plasma cells (Table 1). Amplification of chromosome 1q, which contains the BCL9 locus, is recurrently observed in MM. However, 1q gain did not significantly correlate to *BCL9* mRNA expression, suggesting that other mechanisms drive BCL9 expression [72, 78]. Indeed, loss of microRNA miR30–5p, which targets the 3′UTR of *BCL9*, has recently been identified as important mechanism underlying BCL9 overexpression in malignant plasma cells [79]. In functional studies, shRNA-mediated knockdown of BCL9 in HMCLs suppressed Wnt signaling and attenuated tumor growth in vitro and in vivo. In addition, BCL9 silencing decreased the migration of tumor cells and suppressed expression of vascular endothelial growth factor (VEGF) [79]. Similar results were obtained by suppressing BCL9 function with ectopic expression of miR-30–5p [79] or treatment with a BCL9-derived peptide that specifically disrupts the BCL9/β–catenin interaction [80], supporting the notion that BCL9 overexpression mediates Wnt-driven tumorigenesis in MM. In addition to BCL9, mucin1-C (MUC1-C) was recently also reported to act as transcriptional co-activator in Wnt signaling, increasing β-catenin occupancy on the MYC promoter and forming a complex with β-catenin and TCF4, thereby driving MYC transcription [81].

Collectively, the above-described studies support a model in which loss of Wnt antagonists or aberrant expression of positive Wnt regulators sensitizes MM cells to autocrine and paracrine Wnt ligands. This results in hyperactivation of canonical Wnt signaling and enhances proliferation, migration, and drug resistance of MM cells.

## Wnt signaling in MM bone disease

Osteolytic lesions are present in the vast majority of MM patients and are frequently accompanied by highly debilitating symptoms such as bone pain and pathological fractures. Current evidence indicates that MM cells induce lytic bone disease by secreting paracrine factors that both instigate osteoclastic bone resorption and impair bone formation by attenuating osteoblast differentiation [82].

Canonical Wnt signaling is required for osteoblast development and plays a key role in bone homeostasis. This is illustrated by rare hereditary diseases characterized by increased or decreased bone mass. Loss of the osteocyte-specific secreted Wnt antagonist sclerostin (Van Buchem disease or sclerosteosis) or mutations in the Wnt coreceptor LRP5, which prevent binding of the Wnt antagonists sclerostin and DKK1, have been shown to cause increased bone mass. Conversely, loss-of-function mutations of LRP5 have been identified in osteoporosis-pseudoglioma syndrome (OPPG), which is characterized by decreased bone mass [83]. A landmark study by Tian et al. [84] identified secretion of the Wnt antagonist DKK1 by MM cells as a key mechanism underlying MM bone disease. Gene-expression analysis revealed that most MMs express DKK1, in contrast to normal plasma cells or MGUS. *DKK1* expression strongly correlated with the presence of lytic bone lesions. Functional studies indicated that DKK1-mediated inhibition of canonical Wnt signaling in osteoblast contributes to the development of osteolytic lesions by attenuating osteoblast differentiation [84, 85].

**Fig. 3:**
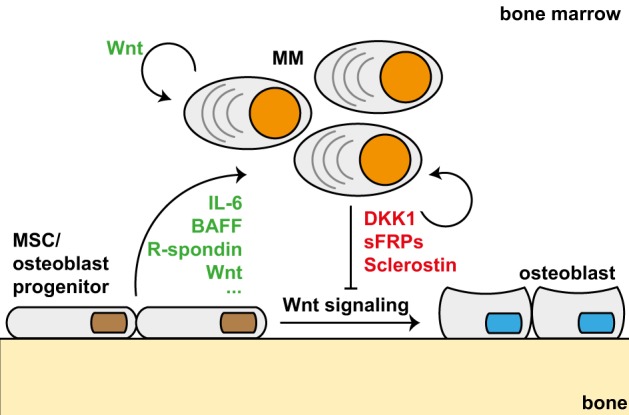
Schematic representation of inhibition of osteoblast differentiation by MM-derived Wnt antagonists. The MM-derived secreted Wnt antagonists DKK1, sFRP, and sclerostin attenuate Wnt signaling in osteoblast precursors, which impairs osteoblasts differentiation and contributes to the development of osteolytic lesions. Inhibition of osteoblast differentiation creates a tumor permissive environment, since osteoblast precursors secrete high levels of IL-6, BAFF, and other cytokines and growth factors. In addition, immature osteoblasts secrete R-spondin, which protects LGR4-expressing MM cells against secreted Wnt antagonists by enhancing sensitivity of tumor cells to autocrine and paracrine Wnt ligands

In addition to DKK1, the secreted Wnt inhibitors sFRP2 [86], sFRP3/FRZB [87, 88] and sclerostin [89, 90] have also been reported to be overexpressed in MM. Of note, only *DKK1* and *sFRP3/FRZB* expression levels were significantly correlated to the presence of lytic bone disease [91]. Intriguingly, expression of DKK1 as well as of other secreted Wnt antagonists is largely restricted to early disease stages. During disease progression, expression of these Wnt antagonists is frequently lost due to promoter methylation. This suggests that Wnt antagonists exert disease stage-specific functions [61, 62, 65]. Following the identification of secreted Wnt antagonists as drivers of lytic bone disease, the therapeutic potential of activating Wnt signaling in osteoblasts was studied in various murine in vivo MM models. Stimulation of Wnt signaling in the tumor microenvironment either by ectopic expression of Wnt3a in HMCLs [54] or by systemic administration of Wnt3a, GSK3-inhibitors [56] or an anti-DKK1 monoclonal antibody (mAb) [92, 93] was found to increase osteoblast numbers, enhance bone formation, and prevent the development of osteolytic lesions. Collectively, these results provided strong evidence for a key role of deregulated Wnt signaling in the development of osteolytic lesions. Interestingly, some studies reported that the increase in bone formation in these models was accompanied by a decrease in tumor burden, which seems to contradict the notion that Wnt activation drives proliferation of MM cells. However, additional experiments revealed that the GSK3 inhibitor LiCl only decreased tumor growth in the BM, while the growth of subcutaneously inoculated MM cells was enhanced [56]. Ectopic expression of dnTCF4 in the MM cells blocked the LiCl-induced growth of subcutaneous tumors, confirming that it was indeed driven by Wnt signaling. Furthermore, ectopic expression of Wnt3a in MM cells or treatment of MM-bearing mice with Wnt3a [54] or with anti-DKK1 mAbs [92, 93] was reported to decrease growth of intramedullary MMs but did not suppress the growth of subcutaneously inoculated MMs. However, in these cases no growth acceleration of subcutaneous tumors was observed. These data suggest that MM-derived Wnt antagonists indirectly enhance tumorigenesis by suppressing osteoblast differentiation. Consistent with this scenario, it was reported that osteoblasts precursors secrete significant higher levels of IL-6, IL-10, BAFF, HGF, and VEGF, suggesting that suppression of osteoblast differentiation enhances tumor growth by creating a tumor permissive microenvironment [55, 92, 94] (Fig. 3). Of note, we recently reported that immature osteoblasts also secrete high levels of R-spondin, which may protect LGR4-expressing MM cells against Wnt inhibition by secreted Wnt antagonist [63].

## Conclusions and therapeutic perspectives

As discussed in this review, current evidence indicates that canonical Wnt signaling plays a dual and disease stage-specific role in the pathogenesis of MM. At early disease stages when MM cells are strictly confined to the BM, they secrete various Wnt antagonists, which attenuate osteoblast differentiation and thereby contribute to the development of osteolytic lesions. Since osteoblast precursors secrete high levels of cytokines and growth factors that promote MM cell growth and survival this creates a tumor permissive environment (Fig. 3). During MM disease progression, the secreted Wnt antagonists are subject to epigenetic silencing, which facilitates Wnt pathway activation and Wnt-driven proliferation of MM cells.

Oncogenic Wnt activity is typically driven by mutations in key Wnt pathway components, but these pathway intrinsic mutations are rare in MM (Table 1). Instead, Wnt pathway activation in MM is largely fueled by autocrine and paracrine Wnt ligands and promoted by epigenetic silencing of secreted Wnt antagonist, overexpression of the co-receptor LGR4 and the transcriptional co-activator BCL9, expression of HSPGs, sumoylation of β-catenin and mutation/deletion of the negative regulator CYLD (Table 1 and Fig. 2). This indicates that illegitimate Wnt activation in MM results from ‘releasing the breaks’ rather than ‘hitting the gas’. Aberrant Wnt pathway activation in MM cells mediates proliferation by inducing expression of cell cycle genes, such as *CCND1, MYC*, and *AURKA* and causes drug resistance. Of note, deregulation of the cell cycle is a unifying pathogenic event required for malignant transformation of plasma cells [4].

Given the role of DKK1 in the development of lytic bone disease, a mAb targeting DKK1 has been developed and has been tested in clinical trials. Initial reported results indicate that anti-DKK1 treatment is well tolerated; thus far effects on MM bone disease and disease outcome have not been reported [95]. Importantly, anti-DKK1 treatment will likely not only increase Wnt signaling in osteoblasts but also in the tumor cells. Furthermore, apart from DKK-1 MM cells express a number of additional Wnt antagonists that will not be targeted using this strategy. Since DKK1 and other Wnt antagonists present bonafide transcriptional feedback targets of Wnt signaling in many tissues, it will be important to determine if they are also transcriptionally regulated by Wnt signaling in MM (see also wnt.stanford.edu) [96–98]. If so, targeting Wnt signaling in MM cells may not only decrease proliferation and drug resistance, but also suppress secretion of Wnt antagonist, thus preventing osteoblast inhibition and MM bone disease.

In addition to canonical Wnt signaling, several studies have indicated a role of non-canonical Wnt signaling in MM pathogenesis. Activation of this signaling cascade involves signaling true RhoA/ROCK and PKC family members, and mediates migration, invasion, and adhesion of MM cells, resulting in tumor dissemination and CAM-DR.

The central role of the Wnt pathway in a wide variety of malignancies has led to the development and (pre)clinical testing of many monoclonal antibodies, and small molecule inhibitors, targeting the Wnt signaling cascade at multiple distinct levels (Fig. 2 and Table 2) [30, 80, 99–109]. Despite the fact that Wnt signaling is essential for many homeostatic functions, these drugs have proven to be surprisingly well tolerated. Importantly, the Wnt signaling cascade in MM is essentially intact and Wnt activation is largely ligand dependent. This implies that Wnt signaling can be targeted by drugs that act on membrane proximal in the pathway, such as Porcupine inhibitors interfering with secretion of Wnts, or mAbs and small molecules/peptides that sequester Wnts or block Wnt receptors. In addition, given its central role in regulating the sensitivity of tumor cells to Wnt ligands, blocking LGR4/R–spondin interaction with mAbs or other compounds is a potentially attractive strategy to target Wnt signaling in MM (Fig. 2 and Table 2). Furthermore, targeting the BCL9/β-catenin complex presents a potentially promising therapeutic strategy to inhibit canonical Wnt signaling in MM cells, since BCL9 is exclusively expressed by malignant plasma cells but not by normal plasma cells and many other cell types [10]. Since most MM display hallmarks of Wnt activation, irrespective of the genetic background, targeting the Wnt pathway is potentially of benefit for a large group of MM patients. Further studies are required to define the best strategy for targeting canonical Wnt signaling in MM, possibly by uncoupling Wnt signaling in MM cells from Wnt activation in the BM niche.

**Table 2:** Overview of monoclonal antibodies, peptides, and small molecule inhibitors that target canonical Wnt signaling

Name	Target	Study phase	Trial number
*Monoclonal antibodies/peptides*			
Vantictumab (OMP-18R5) [99]	Frizzled receptors	Phase I	NCT01973309, NCT01345201, NCT02005315, NCT0195700784
Ipafricept (OMP-54F28) [100]	Wnt ligands (Fzd8-Fc)	Phase I	NCT02092363, NCT02050178, NCT02069145, NCT01608867
Rosmantuzumab (OMP-131R10) [101]	R-spondin 3	Phase I	NCT02482441
SAH-BCL9 [80]	BCL9/β-catenin interaction	Preclinical	
*Small molecules*			
LGK974 [30, 102]	Porcupine (Wnt secretion)	Phase I	NCT01351103, NCT02278133
ETC-159 [103]	Porcupine (Wnt secretion)	Phase I	NCT02521844
C59 [104]	Porcupine (Wnt secretion)	Preclinical	
IWP-2 [105]	Porcupine (Wnt secretion)	Preclinical	
XAV939 [106]	Tankyrase (Axin stabilization)	Preclinical	
IWR [105]	Tankyrase (Axin stabilization)	Preclinical	
G007/G244-LM [97]	Tankyrase (Axin stabilization)	Preclinical	
Pyrvinium [40]	CK1α activation	Preclinical^a^	
BC2059 [37]	β-catenin	Phase I	NCT03459469
PKF115–584 [35]	TCF/β–catenin interaction	Preclinical	
ICG-001 [108]	CBP/β–catenin interaction	Preclinical	
PRI-724 [109]	CBP/β–catenin interaction	Phase I/II	NCT01764477, NCT01606579
AV-65 [36]	Unknown	Preclinical	
CGK012 [38]	Unknown	Preclinical	

^a^Pyrvinium is FDA-approved as an anti-helminthic drug

## References

[1] Palumbo A, Anderson K (2011). Multiple myeloma. N Engl J Med.

[2] Manier S, Salem KZ, Park J, Landau DA, Getz G, Ghobrial IM (2017). Genomic complexity of multiple myeloma and its clinical implications. Nat Rev Clin Oncol.

[3] Morgan GJ, Walker BA, Davies FE (2012). The genetic architecture of multiple myeloma. Nat Rev Cancer.

[4] Bergsagel PL, Kuehl WM, Zhan F, Sawyer J, Barlogie B, Shaughnessy J (2005). Cyclin D dysregulation: an early and unifying pathogenic event in multiple myeloma. Blood.

[5] Derksen PW, Tjin E, Meijer HP, Klok MD, MacGillavry HD, van Oers MH (2004). Illegitimate WNT signaling promotes proliferation of multiple myeloma cells. Proc Natl Acad Sci USA.

[6] Hideshima T, Mitsiades C, Tonon G, Richardson PG, Anderson KC (2007). Understanding multiple myeloma pathogenesis in the bone marrow to identify new therapeutic targets. Nat Rev Cancer.

[7] Kawano Y, Moschetta M, Manier S, Glavey S, Görgün GT, Roccaro AM (2015). Targeting the bone marrow microenvironment in multiple myeloma. Immunol Rev.

[8] Chen Z, Orlowski RZ, Wang M, Kwak L, McCarty N (2014). Osteoblastic niche supports the growth of quiescent multiple myeloma cells. Blood.

[9] Reijmers RM, Spaargaren M, Pals ST (2013). Heparan sulfate proteoglycans in the control of B cell development and the pathogenesis of multiple myeloma. FEBS J.

[10] Derksen PW, Keehnen RM, Evers LM, van Oers MH, Spaargaren M, Pals ST (2002). Cell surface proteoglycan syndecan-1 mediates hepatocyte growth factor binding and promotes Met signaling in multiple myeloma. Blood.

[11] Sanderson RD, Yang Y (2008). Syndecan-1: a dynamic regulator of the myeloma microenvironment. Clin Exp Metastas-.

[12] Clevers H, Loh KM, Nusse R (2014). Stem cell signaling. An integral program for tissue renewal and regeneration: Wnt signaling and stem cell control. Science.

[13] Nusse R, Clevers H (2017). Wnt/β-catenin signaling, disease, and emerging therapeutic modalities. Cell.

[14] Willert K, Brown JD, Danenberg E, Duncan AW, Weissman IL, Reya T (2003). Wnt proteins are lipid-modified and can act as stem cell growth factors. Nature.

[15] Kadowaki T, Wilder E, Klingensmith J, Zachary K, Perrimon N (1996). The segment polarity gene porcupine encodes a putative multitransmembrane protein involved in Wingless processing. Genes Dev.

[16] Behrens J, von Kries JP, Kühl M, Bruhn L, Wedlich D, Grosschedl R (1996). Functional interaction of beta-catenin with the transcription factor LEF-1. Nature.

[17] Molenaar M, van de Wetering M, Oosterwegel M, Peterson-Maduro J, Godsave S, Korinek V (1996). XTcf-3 transcription factor mediates beta-catenin-induced axis formation in *Xenopus* embryos. Cell.

[18] Kramps T, Peter O, Brunner E, Nellen D, Froesch B, Chatterjee S (2002). Wnt/wingless signaling requires BCL9/legless-mediated recruitment of pygopus to the nuclear beta-catenin-TCF complex. Cell.

[19] He TC, Sparks AB, Rago C, Hermeking H, Zawel L, da Costa LT (1998). Identification of c-MYC as a target of the APC pathway. Science.

[20] Tetsu O, McCormick F (1999). Beta-catenin regulates expression of cyclin D1 in colon carcinoma cells. Nature.

[21] Fujisawa K, Fujita A, Ishizaki T, Saito Y, Narumiya S (1996). Identification of the Rho-binding domain of p160ROCK, a Rho-associated coiled-coil containing protein kinase. J Biol Chem.

[22] Klingensmith J, Nusse R, Perrimon N (1994). The *Drosophila* segment polarity gene dishevelled encodes a novel protein required for response to the wingless signal. Genes Dev.

[23] Strutt DI, Weber U, Mlodzik M (1997). The role of RhoA in tissue polarity and frizzled signalling. Nature.

[24] Kühl M, Sheldahl LC, Park M, Miller JR, Moon RT (2000). The Wnt/Ca2+pathway: a new vertebrate Wnt signaling pathway takes shape. Trends Genet.

[25] Clevers H, Nusse R (2012). Wnt/beta-catenin signaling and disease. Cell.

[26] de Lau W, Barker N, Low TY, Koo BK, Li VS, Teunissen H (2011). Lgr5 homologues associate with Wnt receptors and mediate R-spondin signalling. Nature.

[27] Carmon KS, Gong X, Lin Q, Thomas A, Liu Q (2011). R-spondins function as ligands of the orphan receptors LGR4 and LGR5 to regulate Wnt/beta-catenin signaling. Proc Natl Acad Sci USA.

[28] Hao HX, Xie Y, Zhang Y, Charlat O, Oster E, Avello M (2012). ZNRF3 promotes Wnt receptor turnover in an R-spondin-sensitive manner. Nature.

[29] Koo BK, Spit M, Jordens I, Low TY, Stange DE, van de Wetering M (2012). Tumour suppressor RNF43 is a stem-cell E3 ligase that induces endocytosis of Wnt receptors. Nature.

[30] Jiang X, Hao HX, Growney JD, Woolfenden S, Bottiglio C, Ng N (2013). Inactivating mutations of RNF43 confer Wnt dependency in pancreatic ductal adenocarcinoma. Proc Natl Acad Sci USA.

[31] Wu J, Jiao Y, Dal Molin M, Maitra A, de Wilde RF, Wood LD (2011). Whole-exome sequencing of neoplastic cysts of the pancreas reveals recurrent mutations in components of ubiquitin-dependent pathways. Proc Natl Acad Sci USA.

[32] Seshagiri S, Stawiski EW, Durinck S, Modrusan Z, Storm EE, Conboy CB (2012). Recurrent R-spondin fusions in colon cancer. Nature.

[33] Ashihara E, Kawata E, Nakagawa Y, Shimazaski C, Kuroda J, Taniguchi K (2009). Beta-catenin small interfering RNA successfully suppressed progression of multiple myeloma in a mouse model. Clin Cancer Res.

[34] Dutta-Simmons J, Zhang Y, Gorgun G, Gatt M, Mani M, Hideshima T (2009). Aurora kinase A is a target of Wnt/beta-catenin involved in multiple myeloma disease progression. Blood.

[35] Sukhdeo K, Mani M, Zhang Y, Dutta J, Yasui H, Rooney MD (2007). Targeting the beta-catenin/TCF transcriptional complex in the treatment of multiple myeloma. Proc Natl Acad Sci USA.

[36] Yao H, Ashihara E, Strovel JW, Nakagawa Y, Kuroda J, Nagao R (2011). AV-65, a novel Wnt/β-catenin signal inhibitor, successfully suppresses progression of multiple myeloma in a mouse model. Blood Cancer J.

[37] Savvidou I, Khong T, Cuddihy A, McLean C, Horrigan S, Spencer A (2017). β-catenin inhibitor BC2059 is efficacious as monotherapy or in combination with proteasome inhibitor bortezomib in multiple myeloma. Mol Cancer Ther.

[38] Choi PJ, OY, Her JH, Yun E, Song GY, Oh (2017). Anti-proliferative activity of CGK012 against multiple myeloma cells via Wnt/β-catenin signaling attenuation. Leuk Res.

[39] Thorne CA, Hanson AJ, Schneider J, Tahinci E, Orton D, Cselenyi CS (2010). Small-molecule inhibition of Wnt signaling through activation of casein kinase 1α. Nat Chem Biol.

[40] Xu F, Zhu Y, Lu Y, Yu Z, Zhong J, Li Y (2018). Anthelmintic pyrvinium pamoate blocks Wnt/β-catenin and induces apoptosis in multiple myeloma cells. Oncol Lett.

[41] Bjorklund CC, Ma W, Wang ZQ, Davis RE, Kuhn DJ, Kornblau SM (2011). Evidence of a role for activation of Wnt/beta-catenin signaling in the resistance of plasma cells to lenalidomide. J Biol Chem.

[42] Dechow T, Steidle S, Götze KS, Rudelius M, Behnke K, Pechloff K (2014). GP130 activation induces myeloma and collaborates with MYC. J Clin Invest.

[43] Selvanayagam P, Blick M, Narni F, van Tuinen P, Ledbetter DH, Alexanian R (1988). Alteration and abnormal expression of the c-myc oncogene in human multiple myeloma. Blood.

[44] Tao H, Shinmura K, Yamada H, Maekawa M, Osawa S, Takayanagi Y (2010). Identification of 5 novel germline APC mutations and characterization of clinical phenotypes in Japanese patients with classical and attenuated familial adenomatous polyposis. BMC Res Notes.

[45] Mulligan G, Lichter DI, Di Bacco A, Blakemore SJ, Berger A, Koenig E (2014). Mutation of NRAS but not KRAS significantly reduces myeloma sensitivity to single-agent bortezomib therapy. Blood.

[46] Lohr JG, Stojanov P, Carter SL, Cruz-Gordillo P, Lawrence MS, Auclair D (2014). Widespread genetic heterogeneity in multiple myeloma: implications for targeted therapy. Cancer Cell.

[47] Bolli N, Avet-Loiseau H, Wedge DC, Van Loo P, Alexandrov LB, Martincorena I (2014). Heterogeneity of genomic evolution and mutational profiles in multiple myeloma. Nat Commun.

[48] Bolli N, Li Y, Sathiaseelan V, Raine K, Jones D, Ganly P (2016). A DNA target-enrichment approach to detect mutations, copy number changes and immunoglobulin translocations in multiple myeloma. Blood Cancer J.

[49] Qiang YW, Endo Y, Rubin JS, Rudikoff S (2003). Wnt signaling in B-cell neoplasia. Oncogene.

[50] Qiang YW, Walsh K, Yao L, Kedei N, Blumberg PM, Rubin JS (2005). Wnts induce migration and invasion of myeloma plasma cells. Blood.

[51] Kobune M, Chiba H, Kato J, Kato K, Nakamura K, Kawano Y (2007). Wnt3/RhoA/ROCK signaling pathway is involved in adhesion-mediated drug resistance of multiple myeloma in an autocrine mechanism. Mol Cancer Ther.

[52] Reya T, O’Riordan M, Okamura R, Devaney E, Willert K, Nusse R (2000). Wnt signaling regulates B lymphocyte proliferation through a LEF-1 dependent mechanism. Immunity.

[53] Reya T, Duncan AW, Ailles L, Domen J, Scherer DC, Willert K (2003). A role for Wnt signalling in self-renewal of haematopoietic stem cells. Nature.

[54] Qiang YW, Shaughnessy JD, Yaccoby S (2008). Wnt3a signaling within bone inhibits multiple myeloma bone disease and tumor growth. Blood.

[55] Gunn WG, Conley A, Deininger L, Olson SD, Prockop DJ, Gregory CA (2006). A crosstalk between myeloma cells and marrow stromal cells stimulates production of DKK1 and interleukin-6: a potential role in the development of lytic bone disease and tumor progression in multiple myeloma. Stem Cells.

[56] Gunn WG, Krause U, Lee N, Gregory CA (2011). Pharmaceutical inhibition of glycogen synthetase kinase-3β reduces multiple myeloma-induced bone disease in a novel murine plasmacytoma xenograft model. Blood.

[57] Cohen P, Frame S (2001). The renaissance of GSK3. Nat Rev Mol Cell Biol.

[58] Albuquerque C, Breukel C, van der Luijt R, Fidalgo P, Lage P, Slors FJ (2002). The ‘just-right’ signaling model: APC somatic mutations are selected based on a specific level of activation of the beta-catenin signaling cascade. Hum Mol Genet.

[59] Christie M, Jorissen RN, Mouradov D, Sakthianandeswaren A, Li S, Day F (2013). Different APC genotypes in proximal and distal sporadic colorectal cancers suggest distinct WNT/β-catenin signalling thresholds for tumourigenesis. Oncogene.

[60] Luis TC, Ichii M, Brugman MH, Kincade P, Staal FJ (2012). Wnt signaling strength regulates normal hematopoiesis and its deregulation is involved in leukemia development. Leukemia.

[61] Kocemba KA, Groen RW, van Andel H, Kersten MJ, Mahtouk K, Spaargaren M (2012). Transcriptional silencing of the Wnt-antagonist DKK1 by promoter methylation is associated with enhanced Wnt signaling in advanced multiple myeloma. PLoS One.

[62] Chim CS, Pang R, Fung TK, Choi CL, Liang R (2007). Epigenetic dysregulation of Wnt signaling pathway in multiple myeloma. Leukemia.

[63] van Andel H, Ren Z, Koopmans I, Joosten SP, Kocemba KA, de Lau W (2017). Aberrantly expressed LGR4 empowers Wnt signaling in multiple myeloma by hijacking osteoblast-derived R-spondins. Proc Natl Acad Sci USA.

[64] Gonzalez-Paz N, Chng WJ, McClure RF, Blood E, Oken MM, Van Ness B (2007). Tumor suppressor p16 methylation in multiple myeloma: biological and clinical implications. Blood.

[65] Jost E, Gezer D, Wilop S, Suzuki H, Herman JG, Osieka R (2009). Epigenetic dysregulation of secreted frizzled-related proteins in multiple myeloma. Cancer Lett.

[66] Ren Z, van Andel H, de Lau W, Hartholt RB, Maurice MM, Clevers H (2018). Syndecan-1 promotes Wnt/β-catenin signaling in multiple myeloma by presenting Wnts and R-spondins. Blood.

[67] Reijmers RM, Groen RW, Rozemuller H, Kuil A, de Haan-Kramer A, Csikós T (2010). Targeting EXT1 reveals a crucial role for heparan sulfate in the growth of multiple myeloma. Blood.

[68] Walker BA, Leone PE, Chiecchio L, Dickens NJ, Jenner MW, Boyd KD (2010). A compendium of myeloma-associated chromosomal copy number abnormalities and their prognostic value. Blood.

[69] Walker BA, Boyle EM, Wardell CP, Murison A, Begum DB, Dahir NM (2015). Mutational spectrum, copy number changes, and outcome: results of a sequencing study of patients with newly diagnosed myeloma. J Clin Oncol.

[70] Keats JJ, Fonseca R, Chesi M, Schop R, Baker A, Chng WJ (2007). Promiscuous mutations activate the noncanonical NF-kappaB pathway in multiple myeloma. Cancer Cell.

[71] van Andel H, Kocemba KA, de Haan-Kramer A, Mellink CH, Piwowar M, Broijl A (2017). Loss of CYLD expression unleashes Wnt signaling in multiple myeloma and is associated with aggressive disease. Oncogene.

[72] Carrasco DR, Tonon G, Huang Y, Zhang Y, Sinha R, Feng B (2006). High-resolution genomic profiles define distinct clinico-pathogenetic subgroups of multiple myeloma patients. Cancer Cell.

[73] Brummelkamp TR, Nijman SM, Dirac AM, Bernards R (2003). Loss of the cylindromatosis tumour suppressor inhibits apoptosis by activating NF-kappaB. Nature.

[74] Kovalenko A, Chable-Bessia C, Cantarella G, Israël A, Wallach D, Courtois G (2003). The tumour suppressor CYLD negatively regulates NF-kappaB signalling by deubiquitination. Nature.

[75] Tauriello DV, Haegebarth A, Kuper I, Edelmann MJ, Henraat M, Canninga-van Dijk MR (2010). Loss of the tumor suppressor CYLD enhances Wnt/beta-catenin signaling through K63-linked ubiquitination of Dvl. Mol Cell.

[76] Huang HJ, Zhou LL, Fu WJ, Zhang CY, Jiang H, Du J (2014). β-catenin SUMOylation is involved in the dysregulated proliferation of myeloma cells. Am J Cancer Res.

[77] Driscoll JJ, Pelluru D, Lefkimmiatis K, Fulciniti M, Prabhala RH, Greipp PR (2010). The sumoylation pathway is dysregulated in multiple myeloma and is associated with adverse patient outcome. Blood.

[78] Mani M, Carrasco DE, Zhang Y, Takada K, Gatt ME, Dutta-Simmons J (2009). BCL9 promotes tumor progression by conferring enhanced proliferative, metastatic, and angiogenic properties to cancer cells. Cancer Res.

[79] Zhao JJ, Lin J, Zhu D, Wang X, Brooks D, Chen M (2014). miR-30-5p functions as a tumor suppressor and novel therapeutic tool by targeting the oncogenic Wnt/β-catenin/BCL9 pathway. Cancer Res.

[80] Takada K, Zhu D, Bird GH, Sukhdeo K, Zhao JJ, Mani M (2012). Targeted disruption of the BCL9/β-catenin complex inhibits oncogenic Wnt signaling. Sci Transl Med.

[81] Tagde A, Rajabi H, Bouillez A, Alam M, Gali R, Bailey S (2016). MUC1-C drives MYC in multiple myeloma. Blood.

[82] O’Donnell EK, Raje NS (2017). Myeloma bone disease: pathogenesis and treatment. Clin Adv Hematol Oncol.

[83] Baron R, Kneissel M (2013). WNT signaling in bone homeostasis and disease: from human mutations to treatments. Nat Med.

[84] Tian E, Zhan F, Walker R, Rasmussen E, Ma Y, Barlogie B (2003). The role of the Wnt-signaling antagonist DKK1 in the development of osteolytic lesions in multiple myeloma. N Engl J Med.

[85] Heath DJ, Chantry AD, Buckle CH, Coulton L, Shaughnessy JD, Evans HR (2009). Inhibiting Dickkopf-1 (Dkk1) removes suppression of bone formation and prevents the development of osteolytic bone disease in multiple myeloma. J Bone Miner Res.

[86] Oshima T, Abe M, Asano J, Hara T, Kitazoe K, Sekimoto E (2005). Myeloma cells suppress bone formation by secreting a soluble Wnt inhibitor, sFRP-2. Blood.

[87] Davies FE, Dring AM, Li C, Rawstron AC, Shammas MA, O’Connor SM (2003). Insights into the multistep transformation of MGUS to myeloma using microarray expression analysis. Blood.

[88] De Vos J, Couderc G, Tarte K, Jourdan M, Requirand G, Delteil MC (2001). Identifying intercellular signaling genes expressed in malignant plasma cells by using complementary DNA arrays. Blood.

[89] Colucci S, Brunetti G, Oranger A, Mori G, Sardone F, Specchia G (2011). Myeloma cells suppress osteoblasts through sclerostin secretion. Blood Cancer J.

[90] McDonald MM, Reagan MR, Youlten SE, Mohanty ST, Seckinger A, Terry RL (2017). Inhibiting the osteocyte-specific protein sclerostin increases bone mass and fracture resistance in multiple myeloma. Blood.

[91] Kristensen IB, Christensen JH, Lyng MB, Møller MB, Pedersen L, Rasmussen LM (2014). Expression of osteoblast and osteoclast regulatory genes in the bone marrow microenvironment in multiple myeloma: only up-regulation of Wnt inhibitors SFRP3 and DKK1 is associated with lytic bone disease. Leuk Lymphoma.

[92] Fulciniti M, Tassone P, Hideshima T, Vallet S, Nanjappa P, Ettenberg SA (2009). Anti-DKK1 mAb (BHQ880) as a potential therapeutic agent for multiple myeloma. Blood.

[93] Yaccoby S, Ling W, Zhan F, Walker R, Barlogie B, Shaughnessy JD (2007). Antibody-based inhibition of DKK1 suppresses tumor-induced bone resorption and multiple myeloma growth in vivo. Blood.

[94] Reagan MR, Ghobrial IM (2012). Multiple myeloma mesenchymal stem cells: characterization, origin, and tumor-promoting effects. Clin Cancer Res.

[95] Iyer SP, Beck JT, Stewart AK, Shah J, Kelly KR, Isaacs R (2014). A Phase IB multicentre dose-determination study of BHQ880 in combination with anti-myeloma therapy and zoledronic acid in patients with relapsed or refractory multiple myeloma and prior skeletal-related events. Br J Haematol.

[96] Niida A, Hiroko T, Kasai M, Furukawa Y, Nakamura Y, Suzuki Y (2004). DKK1, a negative regulator of Wnt signaling, is a target of the beta-catenin/TCF pathway. Oncogene.

[97] González-Sancho JM, Aguilera O, García JM, Pendás-Franco N, Peña C, Cal S (2005). The Wnt antagonist DICKKOPF-1 gene is a downstream target of beta-catenin/TCF and is downregulated in human colon cancer. Oncogene.

[98] Chamorro MN, Schwartz DR, Vonica A, Brivanlou AH, Cho KR, Varmus HE (2005). FGF-20 and DKK1 are transcriptional targets of beta-catenin and FGF-20 is implicated in cancer and development. EMBO J.

[99] Gurney A, Axelrod F, Bond CJ, Cain J, Chartier C, Donigan L (2012). Wnt pathway inhibition via the targeting of Frizzled receptors results in decreased growth and tumorigenicity of human tumors. Proc Natl Acad Sci USA.

[100] Jimeno A, Gordon M, Chugh R, Messersmith W, Mendelson D, Dupont J (2017). A first-in-human Phase I study of the anticancer stem cell agent ipafricept (OMP-54F28), a decoy receptor for Wnt ligands, in patients with advanced solid tumors. Clin Cancer Res.

[101] Katoh M, Katoh M (2017). Molecular genetics and targeted therapy of WNT-related human diseases (review). Int J Mol Med.

[102] Liu J, Pan S, Hsieh MH, Ng N, Sun F, Wang T (2013). Targeting Wnt-driven cancer through the inhibition of porcupine by LGK974. Proc Natl Acad Sci Usa.

[103] Madan B, Ke Z, Harmston N, Ho SY, Frois AO, Alam J (2016). Wnt addiction of genetically defined cancers reversed by PORCN inhibition. Oncogene.

[104] Proffitt KD, Madan B, Ke Z, Pendharkar V, Ding L, Lee MA (2013). Pharmacological inhibition of the Wnt acyltransferase PORCN prevents growth of WNT-driven mammary cancer. Cancer Res.

[105] Chen B, Dodge ME, Tang W, Lu J, Ma Z, Fan CW (2009). Small molecule-mediated disruption of Wnt-dependent signaling in tissue regeneration and cancer. Nat Chem Biol.

[106] Huang SM, Mishina YM, Liu S, Cheung A, Stegmeier F, Michaud GA (2009). Tankyrase inhibition stabilizes axin and antagonizes Wnt signalling. Nature.

[107] Lau T, Chan E, Callow M, Waaler J, Boggs J, Blake RA (2013). A novel tankyrase small-molecule inhibitor suppresses APC mutation-driven colorectal tumor growth. Cancer Res.

[108] Emami KH, Nguyen C, Ma H, Kim DH, Jeong KW, Eguchi M (2004). A small molecule inhibitor of beta-catenin/CREB-binding protein transcription [corrected]. Proc Natl Acad Sci USA.

[109] Lenz HJ, Kahn M (2014). Safely targeting cancer stem cells via selective catenin coactivator antagonism. Cancer Sci.

